# Pre-service factors associated with sexual misconduct among male U.S. Marines

**DOI:** 10.1371/journal.pone.0278640

**Published:** 2022-12-09

**Authors:** Cynthia A. LeardMann, Yohannes G. Haile, Jennifer McAnany, Valerie A. Stander, Diane Williams, Jeffrey Millegan, Keyia N. Carlton, for the Recruit Assessment Program

**Affiliations:** 1 Leidos, San Diego, California, United States of America; 2 Deployment Health Research Department, Naval Health Research Center, San Diego, California, United States of America; 3 Warfighter Performance Research Department, Naval Health Research Center, San Diego, California, United States of America; 4 Uniformed Services University of the Health Sciences, Bethesda, Maryland, United States of America; Jordan University of Science and Technology, JORDAN

## Abstract

**Purpose:**

Sexual assault is a prevalent and persistent problem in the military, yet few studies have examined predictors of sexual offenses. The study aim was to determine pre-service factors associated with sexual offense conviction among U.S. Marines.

**Methods:**

This retrospective cohort study analyzed data from male active duty U.S. Marines (2003–2018). Pre-service factors were assessed using survey data from the Recruit Assessment Program, obtained prior to recruit training at the Marine Corps Recruit Depot, San Diego, California. These survey data were linked with sexual offense conviction data obtained from the Naval Criminal Investigative Service Consolidated Law Enforcement Operations Center.

**Results:**

Of the 146,307 participants, the majority were 18–19 years old (66.7%) and non-Hispanic, White (62.1%) with a high school education or less (76.8%); 107 received convictions for a sexual offense. In unadjusted analyses, race and ethnicity, parental education, type of primary caregiver, parental death, family economic status, childhood emotional trauma, childhood physical abuse, childhood sexual abuse, and unprotected sex were associated with a sexual offense conviction. In the final multivariable model, race and ethnicity (American Indian/Alaskan Native, odds ratio [OR]: 5.28, 95% confidence interval [CI]: 1.86–14.98; Hispanic, OR: 1.83, 95% CI: 1.06–3.18; multiracial/other, OR: 3.28, 95% CI: 1.56–6.89), education (≤ high school, OR: 2.65; 95% CI: 1.21–5.80), parental death (OR: 2.27; 95% CI: 1.16–4.45), unprotected sex (OR: 1.78; 95% CI: 1.03–3.05), and school suspension/expulsion (OR: 1.64; 95% CI: 1.02–2.65) were significant predictors of a subsequent sexual offense conviction.

**Conclusions:**

Results underscore the importance of understanding factors associated with sexual offense and highlight the large discrepancy between self-reported estimates of sexual assault and sexual offense convictions. Findings may inform the development of effective strategies to reduce sexual misconduct, such as technology-facilitated programs that provide private, targeted education; supportive assistance; and prevention materials to individuals who may have elevated sexual misconduct risk.

## Introduction

Sexual assault is increasingly recognized as a major public health concern within the military [1]. Prevention of these type of crimes is a high priority for the Department of Defense (DoD) [2] and thus, changes have been implemented [3]. Since 2005, sexual assault survivors have had two reporting options: a confidential restricted report, or an unrestricted report, which triggers an official investigation [3]. After making either type of report, a range of services, including support and treatment, are available to survivors. There has also been reform to the Uniformed Code of Military Justice, such as the National Defense Authorization Act of Fiscal Year 14, which eliminated the 5-year statute of limitations for violations of sexual assault and removed Commanders’ ability to consider the character and military service of the accused when determining disposition of the offence [4]. The DoD has also worked to improve training for those responsible for responding to sex crimes (e.g., military leadership, criminal investigators, Sexual Assault Response Coordinators) [3].

Despite these changes, rates of sexual assault remain high in the military and are similar to [6] or higher than rates of sexual assault in civilian populations. Based on the DoD Annual Report on Sexual Assault, there were 6,290 official reports of adult sexual assault by U.S. active duty service members in 2020; the Marine Corps had 5.9 reports per 1,000 service members, the highest rate of all the services [5]. The number of official reports of sexual assault in the military has been steadily increasing in the last decade, which may reflect both greater willingness to report as well as an actual increase in prevalence. Still, total reported incidents substantially underestimate the scope of the problem [5, 6]. The discrepancy between sexual assault experienced, and reported sexual assault cases, is a common phenomenon observed in both civilian and military populations, and further complicates efforts to mitigate these crimes.

The number of military personnel experiencing sexual victimization is particularly concerning given that sexual assault not only increases the risk of adverse short- and long-term health and economic outcomes, but also can impede mission readiness and command morale [1, 7]. Research has linked sexual trauma victimization to increased risk of substance abuse, anxiety, eating disorders, posttraumatic stress disorder, early separation from military service, and post-service unemployment [8–11]. However, the majority of research to-date, has examined either impacts of military sexual violence on survivors, or factors associated with increased risk of sexual victimization. In contrast, limited research has focused on the characteristics of military perpetrators of sexual assault [12]. Better understanding of the characteristics and behaviors of perpetrators of military sexual violence is essential in developing effective prevention programs, and providing additional training or intervention strategies to potential offenders, given that they are responsible for sexual violence.

While evidence suggests sexual perpetrators do not share a single set of traits, previous civilian research has identified an array of predictive characteristics associated with sexual offending [13]. For instance, sexual offenders report poor intimacy skills [14, 15]; deviant sexual interests or preferences [15]; and high levels of social difficulties, avoidance, and loneliness [15–18]. Sexual perpetrators also tend to have lifestyle instability and impulse control issues [13, 15], pro-offending attitudes and behaviors [19], and a poor capacity for victim empathy [20]. Other studies suggest a history of impersonal or risky sexual behavior (e.g., early sexual initiation, sex without a condom) heightens risk for sexual aggression [21–23]. In addition, prior delinquent behavior and cumulative exposure to stress or traumatic events predict future sexual aggression [24–26].

Despite the number of previous studies that have examined the characteristics of civilian sexual offenders, research examining characteristics of military sexual perpetrators is sparse [26–28]. One longitudinal study of male U.S. Navy recruits found 13% self-reported perpetrating sexual assault, although the majority (71%) only perpetrated prior to military service [27]. Service men who reported pre-service sexual assault were nearly 10 times more likely to perpetrate during their first year in the Navy. A follow-on study found that a similar set of risk factors predicted sexual harassment and sexual assault [26]. Importantly, researchers found prior sexual assault perpetration, number of sex partners (impersonal sex), hostility to women, and delinquency/misconduct were predictive of sexual harassment and sexual assault. In another study, researchers reviewed administrative data sources of U.S. soldiers and found that sociodemographic, history of deployment, lower rank, criminal history, substance abuse, and mental health factors predicted sexual assault perpetration [28]. Given the limited number of studies, more research is needed to better understand characteristics associated with sexual misconduct among U.S. Marines.

Each year, the Naval Criminal Investigative Service (NCIS) Criminal Data Analysis Division reports data on five major crime topics, including adult sex crimes and child sexual abuse, committed within the nexus of the Department of the Navy, including active duty U.S. Sailors and Marines [29, 30]. Common characteristics of perpetrators of adult sexual assault include being male, active duty, and in the ranks E3 to E6. However, these official reports only provide details of the offenses, victims, and subjects. Thus, additional analyses are needed to compare factors, such as family history, childhood factors, and life stressors, of military perpetrators with the population at large to better understand military sexual offenders. Furthermore, using self-reported data to predict subsequent sexual offense convictions, adjusting for other factors, may provide a unique opportunity to study the effects of pre-service factors.

This retrospective cohort study addressed this knowledge gap by using pre-service survey data collected from a large sample of U.S. Marines, who were participants of the Recruit Assessment Program (RAP), linked with sexual offense conviction data obtained from a military electronic database. The aim was to determine pre-service factors (i.e., demographics, family history, childhood experience, life events and experiences, and risky behaviors) associated with subsequent sexual misconduct among male Marines. Such findings could aid in: 1) developing or redesigning programs to reduce sexual misconduct in the military, 2) identifying patterns of offending behaviors, and 3) assisting investigators in designing interview questions about service members accused of sexual misconduct.

## Materials and methods

### Study design

Since June 2001, the RAP has collected baseline health data on recruits at Marine Corps Recruit Depot (MCRD), San Diego, California. Of the male Marines who enlist and enter active duty, approximately 40% complete basic training at the MCRD in San Diego. Since the inception of RAP, more than 200,000 Marine recruits have voluntarily completed the RAP survey (response rate > 80%). During receiving week prior to recruit training, the RAP survey collects information on demographics, health behaviors, substance use and abuse, family history, childhood experiences, and exposure to traumatic events and psychological stressors. Detailed descriptions of the methodology of the RAP have been described elsewhere [31, 32].

The NCIS Consolidated Law Enforcement Operations Center (CLEOC) serves as a central repository for criminal offenses within the entire Department of the Navy, including the Marine Corps. Specific case information is entered into CLEOC by Department of the Navy law enforcement. A subset of this CLEOC database, Marines with a guilty charge of a sex crime between January 2009 and March 2018, were obtained from NCIS.

For the current study, this subset CLEOC database was linked with self-reported data from active duty male Marines who completed a RAP survey between April 1, 2003 to November 1, 2016 (N = 146,307). The study was approved by the institutional review board at the Naval Health Research Center, and the research was conducted in compliance with all applicable federal regulations governing the protection of human subjects (Protocol NHRC.2000.0003). All participants provided voluntary, informed consent. While some RAP participants were 17 years old, once a person enlists in the military, they are legally considered an adult and no longer considered a dependent minor for most legal purposes. Thus, no consent from their parents or guardians was required for these individuals.

### Patient and public involvement

The research topic for this study was developed from clinicians working with sexual trauma survivors. Knowledge gained from these patient-clinicians relationships indicated that more research should focus on sexual perpetration to better understand patterns of sexual aggression in the DoD and inform policies and practices to hold perpetrators responsible for their sexual violence.

### Outcome

The outcome of interest, sexual offense conviction, was based on a guilty charge of a sex crime as defined in Uniform Code of Military Justice [30] between January 2009 and March 2018, assessed using data from CLEOC. Specifically, these sex crimes included rape, sexual assault, sexual contact (abusive, wrongful, and aggravated), indecent act/exposure/viewing, and sexual abuse.

### Independent predictors

All independent predictors were assessed using self-reported RAP data. The predictors were grouped into five domains: (1) demographics, (2) family history, (3) childhood experiences, (4) life events and experiences, and (5) risky behaviors.

Demographics included three characteristics: age, race and ethnicity, and educational attainment. Family history comprised three variables. Family military history (no or yes) was assessed based on endorsement of either parent serving in the U.S. military. Parent’s education level was categorized into five groups (<high school, high school, some college or associate’s degree, ≥bachelor’s degree, or unknown /not applicable) based on highest education level of either parent who raised the participant. Household smoking (no or yes) was based on positive endorsement of smoking by anyone regularly living in the participant’s household.

Childhood experiences consisted of fourteen indicators. Primary caregiver was categorized into three categories based on responses to the question “Were you mostly raised by (mark all that apply)?” Participants who endorsed “raised by 2 parents” and indicated no other type of caregiver were categorized accordingly. Participants who endorsed “raised by 1 parent” (with or without endorsement of “raised by 2 parents”) and did not endorse any other type of caregiver were categorized accordingly. Those who selected at least one other type of caregiver (e.g., grandparents, foster parents) were classified as raised by other. Parental death (no or yes) was based on reporting the father and/or mother that raised them was no longer alive. Parental separation/divorce (no or yes) was based on the endorsement of a single item. Family economic status (well-off or struggled) was based on reporting of the “family’s ability to provide for your essential needs, such as food, housing, and medical care.” ADHD/learning disability (no or yes) was based on report of a learning disability, ADHD, hyperactivity, or problems paying attention. The remaining childhood experiences were derived from the Adverse Childhood Experiences (ACE) Study [33]. Based on cutoffs used in previous research [33], exposure to physical neglect, emotional neglect, emotional trauma, physical abuse, witnessing domestic violence were each dichotomized (no or yes) based on positive endorsement (“sometimes,” “often,” or “very often”); sexual abuse was dichotomized (no or yes) based on any endorsement (i.e., “once/twice” or more) of childhood sexual assault. Affirmative responses to questions regarding living with someone with depression or mental illness or a drinking problem were used to create two variables (no or yes): guardian with mental health issues and drinking problems, respectively. For a supplemental analysis, an ACE score was created (none, 1, 2, or ≥3) based on the total number of ACE categories reported (i.e., physical neglect, emotional neglect, emotional trauma, physical abuse, sexual abuse, domestic violence exposure, guardian with mental health issues or drinking problems).

Life events and experiences comprised eight indicators. Spiritual involvement was based on one item about attending church, synagogue, or other religious gatherings. Endorsing “about once a week” or “more than once a week” was defined as regular spiritual involvement; all other responses were classified as infrequent. Self-harm was (no or yes) based on a single item (“Have you EVER deliberately cut, burned, or harmed yourself?”). Social isolation was (no or yes) based on reporting having no “close friends or relatives…that you can call on for help or talk to about personal problems.” Losing temper was categorized into three levels based on how often participants reported getting “…mad enough to hit, kick, or throw things” (never, infrequent [once a month or less frequent], frequent [once a week or more]). Fired from job (no or yes) was based on reporting being fired in the year before entering the military. Similarly, history of arrest (no or yes) was assessed based on report of being arrested in the year before entering the military. Being a victim of physical assault (no or yes) was based on those reporting ever being “seriously attacked, beaten up, or assaulted.” Cumulative lifetime trauma exposure was based on exposure to five potentially traumatic events (e.g., threatened with a knife, gun, club, or other weapon; witnessing a stranger being badly injured or killed); the total number of exposures endorsed was summed and collapsed into three groups (none, 1, or ≥2 exposures).

Risky behaviors included eight variables. School suspension/expulsion (no or yes) was based on a single item. For traffic violation, participants were categorized into a 4-level variable (none, 1, 2, or ≥3) based on the number of “traffic tickets for moving violations” ever received. Onset of sexual activity was categorized into a 3-level variable based on age of first sexual intercourse (no sexual intercourse, >15 years, or ≤15 years). Unprotected sex (no or yes) was based on an item about condom use during last sexual encounter; this item was not applicable to those who reported never having sex, so they were placed in a separate category for analytical purposes. Age of smoking onset was determined among those who reported ever smoking at least 100 cigarettes (nonsmoker, >15 years old, or ≤15 years old). Potential alcohol dependence (negative or positive) was determined by endorsement of one or more of the four CAGE (i.e., cut down, annoyance, guilty, eye-opener) items [34]; participants who reported never having an alcohol drink were categorized as negative. Heavy alcohol drinking was created from the standard scoring of the 3-item Alcohol Use Disorders Identification Test-Consumption (AUDIT-C), a tool used to identify hazardous drinking [35]; categorized as nondrinker, moderate drinker [score ≤ 3], and heavy drinker [score >3]. Onset of alcohol drinking (nondrinker, ≥21 years, 16–20 years, ≤15 years) was based on the age reported by recruits in response to the question “Not counting sips, how old were you when you first had a drink containing alcohol?”

### Statistical analyses

Descriptive analyses were performed to compare the predictors by conviction of a sexual offense. Odds ratios (ORs) and 95% confidence intervals (CIs) were estimated using logistic regression. Bivariate logistic regression analyses were performed to assess the relationship of each independent predictor with sexual offense conviction. The multivariable logistic model was derived using a backwards elimination algorithm that initially included all variables significantly associated with sexual offense conviction in the unadjusted models (*p*< .10); this analysis was limited to those with non-missing data for all variables entered into the model (n = 109,956). Independent variables were removed sequentially until the final multivariable model only retained variables significantly associated with the outcome (*p*< .05).

To determine if the results were consistent despite missing data, a supplemental analysis was conducted. Specifically, the final reduced model was repeated including all participants with non-missing data for the variables that remained in the final model. In addition, to examine the impact of cumulative ACEs, we implemented the same procedures as the main multivariable analysis, but replaced the eight individual ACE variables with the ACE score. To examine if the associations between the predictors and sexual offense convictions differed by race or ethnicity (collapsed into a 3-level variable as White non-Hispanic, Hispanic, and other due to small cell sizes), we included interaction terms (race/ethnicity by each predictor) in the initial multivariable model. Multicollinearity was assessed using the variance inflation factor, in which a value of four or greater indicated possible collinearity. All analyses were conducted using SAS/STAT software, version 9.4.

## Results

Of the 146,307 active duty male Marines, the majority were 18–19 years old (n = 97,520, 66.7%) and non-Hispanic, White (n = 90,877, 62.1%) with a high school education or less (n = 112,304, 76.8%; Table 1).

**Table 1:** Demographics, family history, and childhood experiences by sexual offense conviction among U.S. male marines.

	Study sample	No sexual offense conviction		Sexual offense conviction		Unadjusted model	
	N ^a^	n ^a^	%	n ^a^	%	OR	95% CI
Sample	146,307	146,200	99.9	107	0.1	--	--
**Demographics**							
Age*							
17 years	7,726	7,715	5.2	11	10.2	1.90	(1.01–3.59)
18–19 years	97,520	97,447	66.8	73	68.2	1.00	
20–21 years	25,264	25,247	17.2	17	15.8	0.90	(0.53–1.52)
≥22 years	15,672	15,666	10.8	6	5.6	0.51	(0.22–1.18)
Race and ethnicity***							
American Indian/Alaskan Native	2,053	2,046	1.4	7	7.6	7.06	(3.18–15.70)
Asian/Pacific Islander	5,458	5,453	4.0	5	5.4	1.89	(0.75–4.78)
Black non-Hispanic	5,448	5,443	4.0	5	5.4	1.90	(0.75–4.78)
Hispanic	33,968	33,937	24.6	31	33.6	1.89	(1.19–2.99)
White non-Hispanic	90,877	90,833	66.0	44	47.8	1.00	
Multiracial/other	7,574	7,561	5.4	13	14.2	3.55	(1.91–6.59)
Education level*							
≤High school	112,393	112,304	77.4	89	84.8	1.62	(0.95–2.76)
≥Some college	32,763	32,747	22.6	16	15.2	1.00	
**Family History**							
Family military history							
No	108,227	108,142	74.2	85	79.4	1.00	
Yes	37,470	37,448	25.8	22	20.6	0.75	(0.47–1.20)
Household smoking							
No smokers in household	82,759	82,699	59.0	60	58.2	1.00	
Smoker(s) in household	57,492	57,449	41.0	43	41.8	1.03	(0.70–1.53)
Parents educational level**							
<High school	12,178	12,164	8.4	14	13.6	1.99	(1.03–3.84)
High school	38,832	38,799	27.0	33	32.0	1.47	(0.87–2.48)
Some college or associate’s	41,604	41,583	29.0	21	20.4	0.87	(0.49–1.57)
≥Bachelor’s degree	41,447	41,423	28.8	24	23.4	1.00	
Unknown/not applicable	9,499	9,488	6.6	11	10.6	2.00	(0.98–4.09)
**Childhood Experiences**							
Primary caregiver**							
Two parents	98,456	98,400	68.4	56	55.0	1.00	
One parent	33,553	33,517	23.4	36	35.2	1.89	(1.24–2.87)
Other than parent	11,796	11,786	8.2	10	9.8	1.49	(0.76–2.92)
Parental death**							
No	125,934	125,851	93.6	83	87.4	1.00	
Yes	8,577	8,565	6.4	12	12.6	2.13	(1.16–3.90)
Parental separation/divorce							
No	81,111	81,057	57.8	54	53.4	1.00	
Yes	59,425	59,378	42.2	47	46.6	1.19	(0.80–1.76)
Family economic status**							
Well off	90,206	90,154	68.6	52	58.4	1.00	
Struggled	41,480	41,443	31.4	37	41.6	1.55	(1.02–2.36)
ADHD/learning disability							
No	134,747	134,649	93.4	98	94.2	1.00	
Yes	9,645	9,639	6.6	6	5.8	0.86	(0.38–1.95)
Childhood physical neglect							
No	117,102	117,016	84.0	86	84.4	1.00	
Yes	22,449	22,433	16.0	16	15.6	0.97	(0.57–1.66)
Childhood emotional neglect							
No	132,011	131,918	94.8	93	93.0	1.00	
Yes	7,168	7,161	5.2	7	7.0	1.39	(0.64–2.99)
Childhood emotional trauma**							
No	118,354	118,276	85.0	78	77.2	1.00	
Yes	20,855	20,832	15.0	23	22.8	1.67	(1.05–2.67)
Childhood physical abuse**							
No	116,333	116,257	83.6	76	76.0	1.00	
Yes	22,761	22,737	16.4	24	24.0	1.62	(1.02–2.56)
Childhood sexual abuse**							
No	136,107	136,012	98.4	95	95.0	1.00	
Yes	2,276	2,271	1.6	5	5.0	3.15	(1.28–7.76)
Domestic violence exposure*							
No	126,159	126,074	91.0	85	85.8	1.00	
Yes	12,426	12,412	9.0	14	14.2	1.67	(0.95–2.95)
Guardian with mental health issues* issues							
No	126,099	126,015	91.0	84	85.8	1.00	
Yes	12,593	12,579	9.0	14	14.2	1.67	(0.95–2.94)
Guardian with drinking problems							
No	119,934	119,850	86.6	84	84.0	1.00	
Yes	18,716	18,700	13.4	16	16.0	1.22	(0.72–2.08)
ACE score**							
None	79,370	79,320	57.0	50	50.0	1.00	
1	29,169	29,151	20.9	18	18.0	0.98	(0.57–1.68)
2	15,413	15,401	11.1	12	12.0	1.24	(0.66–2.32)
≥3	15,381	15,361	11.0	20	20.0	2.07	(1.23–3.47)

ACE, adverse childhood experience; ADHD, Attention-Deficit/Hyperactivity Disorder; CI, confidence interval; OR, odds ratio.

^a^ Some columns do not sum to the total column n, due to missing data.

^b^ For unprotected sex, those who indicated that they never had sexual intercourse were considered not applicable to answer this question (n = 23,095), but were classified as a separate category (OR: 1.41; 95% CI: 0.86–2.31).

* p-value < .10

** p-value < .05

*** p-value < .01

The life events and experiences as well as risky behaviors are summarized in Table 2. During the study period, 107 (0.07%) Marines in our study sample were convicted of a sexual offense. Most of the convictions were for sexual assault (n = 39, 36.4%) or rape (n = 33, 30.8%), while the remaining were other types of sex crimes, including sexual contact (n = 19, 17.8%), indecent act/exposure/viewing (n = 10, 10.3%), and sexual abuse (n = 5, 4.7%). One third of the victims were children under the age of 16 years (n = 36, 33.6%).

**Table 2:** Life events, experiences and risky behaviors by sexual offense conviction among U.S. male marines.

	Study sample	No sexual offense conviction		Sexual offense conviction		Unadjusted model	
	N ^a^	n ^a^	%	n ^a^	%	OR	95% CI
Sample	146,307	146,200	99.9	107	0.1	--	--
**Life Events and Experiences**							
Spiritual involvement							
Regular	66,832	66,779	47.4	53	51.4	1.00	
Infrequent	74,260	74,210	52.6	50	48.6	0.85	(0.58–1.25)
Self-harm							
No	137,193	137,093	97.6	100	97.0	1.00	
Yes	3,504	3,501	2.4	3	3.0	1.18	(0.37–3.71)
Social isolation							
No	132,923	132,828	94.0	95	91.4	1.00	
Yes	8,350	8,341	6.0	9	8.6	1.51	(0.76–2.99)
Losing temper							
Never	73,012	72,964	53.2	48	47.0	1.00	
Infrequent	55,056	55,010	40.2	46	45.0	1.27	(0.85–1.91)
Frequent	9,078	9,070	6.6	8	7.8	1.34	(0.63–2.84)
Fired from job							
No	131,247	131,153	95.2	94	93.0	1.00	
Yes	6,691	6,684	4.8	7	7.0	1.46	(0.68–3.15)
History of arrest							
No	133,655	133,556	96.8	99	97.0	1.00	
Yes	4,280	4,277	3.2	3	3.0	0.95	(0.30–2.99)
Victim of physical assault							
No	128,981	128,890	93.4	91	91.0	1.00	
Yes	9,238	9,229	6.6	9	9.0	1.38	(0.70–2.74)
Traumatic exposures							
None	79,352	79,298	57.2	54	54.0	1.00	
1	33,055	33,029	23.8	26	26.0	1.16	(0.72–1.85)
≥2	26,540	26,520	19.2	20	20.0	1.11	(0.66–1.85)
**Risky Behaviors**							
School suspension/expulsion*							
No	102,612	102,546	71.2	66	62.8	1.00	
Yes	41,322	41,283	28.8	39	37.2	1.47	(0.99–2.18)
Traffic violation							
None	78,073	78,010	57.0	63	61.8	1.00	
1	24,204	24,184	17.6	20	19.6	1.02	(0.62–1.69)
2	16,478	16,469	12.0	9	8.8	0.68	(0.34–1.36)
≥3	18,298	18,288	13.4	10	9.8	0.68	(0.35–1.32)
Age at first sexual encounter							
No sexual intercourse	22,884	22,863	16.8	21	20.4	1.00	
>15 years	37,687	37,654	27.6	33	32.0	0.95	(0.55–1.65)
≤15 years	75,506	75,457	55.4	49	47.6	0.71	(0.42–1.18)
Unprotected sex^b^**							
No	82,881	82,831	61.0	50	48.6	1.00	
Yes	30,022	29,989	22.0	33	32.0	1.85	(1.17–2.83)
Onset of smoking							
Nonsmoker	107,499	107,419	78.0	80	77.6	1.00	
>15 years	22,084	22,069	16.0	15	14.6	0.91	(0.53–1.58)
≤15 years	8,235	8,227	6.0	8	7.8	1.31	(0.63–2.70)
Potential alcohol dependence							
Negative	125,465	125,371	89.2	94	92.2	1.00	
Positive	15,236	15,228	10.8	8	7.8	0.70	(0.34–1.44)
Heavy alcohol drinking							
Nondrinker	49,204	49,160	35.6	44	42.0	1.00	
Moderate drinker	66,281	66,230	48.0	51	48.6	0.86	(0.58–1.29)
Heavy drinker	22,861	22,851	16.6	10	9.6	0.49	(0.25–0.97)
Onset of alcohol drinking							
Nondrinker	49,204	49,160	34.8	44	42.0	1.00	
≥21 years	5,969	5,962	4.2	7	6.6	1.31	(0.59–2.91)
16–20 years	56,049	56,014	39.6	35	33.4	0.70	(0.45–1.09)
≤15 years	30,022	30,003	21.2	19	18.0	0.71	(0.41–1.21)

CI, confidence interval; OR, odds ratio.

^a^ Some columns do not sum to the total column n, due to missing data.

^b^ For unprotected sex, those who indicated that they never had sexual intercourse were considered not applicable to answer this question (n = 23,095), but were classified as a separate category (OR: 1.41; 95% CI: 0.86–2.31).

* p-value < .10

** p-value < .05

*** p-value < .01.

Based on unadjusted ORs, demographic factors associated with a sexual offense included: 17 years of age (when entering recruit training); American Indian/Alaskan Native, Hispanic, or multiracial/other race and ethnicity; and ≤high school education (Table 1). In regards to family history, participants whose parents earned ≤high school education were proportionally more likely to have a sexual offense conviction. Many of the childhood experiences were associated with greater likelihood of a sexual offense conviction; Marines who reported being raised by one parent, death of a parent, lower family economic status, and some of the ACEs (i.e., emotional trauma, physical abuse, sexual abuse, domestic violence, guardian with mental health problem) were proportionally more likely to be convicted of a sexual offense. None of the life events or experiences were associated with the outcome (Table 2). The risky behaviors associated with a sexual offense were history of suspension from school and having unprotected sex.

The final, reduced multivariable logistic model consisted of a total of 109,956 participants, including 72 with a sexual offense conviction (Table 3). After implementing backward elimination, five variables remained in the final model: race and ethnicity (American Indian/Alaskan Native OR: 5.28; 95% CI, 1.86–14.98; Hispanic OR: 1.83; 95% CI, 1.06–3.18; multiracial/other OR: 3.28; 95% CI, 1.56–6.89), education (≤high school OR: 2.65; 95% CI, 1.21–5.80), parental death (OR: 2.27; 95% CI, 1.16–4.45), unprotected sex (OR: 1.78; 95% CI, 1.03–3.05), and school suspension/expulsion (OR: 1.64; 95% CI, 1.02–2.65). In the supplemental analysis (n = 124,113), including participants with non-missing data for the five predictors in the final model, effect estimates and statistical significance for each predictor were similar except school suspension/expulsion, which was attenuated and not statistically significant (Table 3). When the individual ACE variables were replaced with the ACE score, results remained largely consistent, although school suspension/expulsion was no longer statistically significant. Race/ethnicity did not significantly moderate any of the association between the predictors and sexual offense conviction (all *p*>.05), thus no additional analyses were conducted to further examine racial/ethnic differences.

**Table 3:** Multivariable logistic regression model for sexual offense conviction among U.S. male marines.

	Model 1: Complete case analysis^a^ (n = 109,956)		Model 2: Supplemental model^b^ (n = 124,113)	
Independent predictors	AOR	95% CI	AOR	95% CI
Race and ethnicity				
American Indian/Alaskan Native	5.28	(1.86–14.98)	5.20	(2.05–13.24)
Asian/Pacific Islander	2.05	(0.72–5.83)	2.05	(0.80–5.22)
Black non-Hispanic	1.13	(0.27–4.74)	0.90	(0.22–3.73)
Hispanic	1.83	(1.06–3.18)	1.87	(1.15–3.06)
White non-Hispanic	1.00		1.00	
Multiracial/Other	3.28	(1.56–6.89)	2.98	(1.49–5.96)
Education level				
≤High school	2.65	(1.21–5.80)	2.30	(1.18–4.45)
≥Some college	1.00		1.00	
Parental death				
No	1.00		1.00	
Yes	2.27	(1.16–4.45)	2.18	(1.19–4.03)
Unprotected sex^c^				
No	1.00		1.00	
Yes	1.78	(1.03–3.05)	1.85	(1.14–3.00)
School suspension or expulsion				
No	1.00		1.00	
Yes	1.64	(1.02–2.65)	1.35	(0.87–2.09)

AOR, adjusted odds ratio; CI, confidence interval.

^a^ Variables associated with the outcome (*p* < .10) in bivariate analyses were included in the initial model: age, race and ethnicity, educational level, parental education level, primary caregiver, parental death, family economic status, school suspension or expulsion, childhood emotional trauma, childhood physical abuse, childhood sexual abuse, domestic violence exposure, guardian with mental health issues and unprotected sex. Variables were removed one at a time, based on highest p-value, until all variables were statistically significant with the outcome (*p* < .05).

^b^ This supplemental model was limited to the independent predictors listed in this table; model was conducted with all participants who had non-missing data for these variables.

^c^ For unprotected sex, those who indicated that they never had sexual intercourse were considered not applicable to answer this question, but were classified as a separate category in the main model (OR: 1.85; 95% CI: 1.02–3.35) and supplemental model (OR: 1.73; 95% CI: 1.01–2.97).

## Discussion

This retrospective cohort study identified certain pre-service factors predictive of a sexual offense conviction among U.S. Marines. It expands on previous research that has examined various aspects of sexual trauma within the military context. To our knowledge, this is the first large prospective study of male U.S. Marines to examine pre-service factors associated with subsequent conviction of sexual offense. Of the numerous factors investigated, predictors associated with increased risk of sexual misconduct included: race and ethnicity, education level, parental death, school suspension/expulsion, and unprotected sex in the final multivariable logistic regression model. Results also underscore how few participants were convicted of a sexual offense over a 9-year period (0.01%), highlighting a large discrepancy between self-report estimates of sexual assault perpetration [27] and convictions of sex crimes.

Consistent with previous research, some childhood adversities were associated with a sexual offense conviction. Marines who experienced the death of a parent were more likely to be convicted of a sexual offense. The death of a parent can be one of the most traumatic childhood experiences; parental death is often clustered with other childhood adversities and may lead to additional life stressors [36]. Death of a parent has been found to be associated with a range of adverse outcomes, including mental health problems [37]; Furthermore, some Scandinavian research suggests parental death is associated with perpetrating a violent crime [38]. It may be that loss of a parent impairs the capacity to regulate emotional and behavioral responses to stressors, which increases likelihood to perpetrate violence. Previous research indicates that childhood adversities may contribute to sexual aggression by encouraging hostility toward intimate partners and impersonal sex; however, none of the other adverse childhood experiences were directly associated with a sexual offense conviction [21, 39, 40]. While many of these ACEs, such as childhood sexual abuse and physical abuse, were associated with a sexual offense conviction in our unadjusted analyses, these experiences were no longer associated with the outcome after adjusting for other factors. This may indicate an indirect relationship between childhood adversities and sexual assault perpetration, as has been suggested by past literature [21, 39]. In addition, some evidence suggests that ACEs, which are more prevalent among service members, appear to have less of a long-term impact among male service members compared with male civilians [41].

A history of engaging in risky behaviors often predicts negative outcomes; we found that Marines who reported a history of school suspension or expulsion had elevated odds of having a sexual offense conviction, though not significant in the supplemental models. This finding is consistent with previous research that indicates that prior misconduct or delinquency is associated with sexual assault [26]. Marines who reported unprotected sex had elevated odds of a sexual offense conviction in comparison with those who had sex with a condom, which aligns with prior findings of history of impersonal or risky sexual behavior (e.g., early sexual initiation, sex without a condom) heightening the risk for sexual aggression [21–23, 42]. Specifically, a previous report observed that men with a history of sexual assault perpetration were at increased risk of condom use resistance [23]. Similarly, Frye [42] found that men who perpetrated interpersonal violence were half as likely to have consistent condom use, even after adjusting for other factors.

Evidence suggests that sexual assault is a highly underreported crime with even fewer cases that proceed to the point of charges or prosecution [5]. Thus, the small number of Marines convicted of a sexual offense in this study is not surprising. In both military and civilian contexts, there are complex factors that discourage reporting and provide challenges to prosecution [43]. For instance, sex crimes often occur with no witnesses and under circumstances that make victims embarrassed or fearful of reporting. The military faces additional unique challenges in terms of charging and convicting potential offenders. Up until the end of 2021, commanders were responsible for deciding how to handle alleged sexual offense assailants; they could instigate administrative action, enact nonjudicial punishment, send to a higher authority, or possibly take no action if the victim declined to participate or evidence was insufficient to prosecute [5]. Consequently, sexual assault survivors may be less motivated to report these types of crimes in a system where sex offenders are less likely to be charged and convicted. The majority of adult victims of military sexual offenders are military members [29]. Using 2018 survey data, only 30% of Marines who experienced sexual assault reported it to military authorities [44]. Service members who have experienced sexual assault report numerous barriers to reporting the crime, including, but not limited to: not wanting people to know, concern that nothing will be done, and potential ostracism or retaliation from fellow military members [44, 45].

Because this study relied on military criminal investigative records to assess convictions of sexual offenses, some of the significant effects identified may be influenced by systemic biases in reporting and prosecuting sex crimes rather than differences in military perpetration patterns. For instance, the NCIS annual reports indicate the majority of the officially reported Marine on Marine sex crimes involved Marines of a similar peer group [46], yet a study found 80% of military sexual offenders were of higher rank than their victim [47]. Thus, it is possible that individuals may be less willing to report, charge, and convict a person of a sex crime if the perpetrator is more senior in rank [47]. In the current study, those with ≤high school degree (vs. some college education) and who identified as American Indian/Alaskan Native, Hispanic or multiracial/other (vs. White, non-Hispanic) were more likely to be convicted of a sexual offense. While these results are consistent with one previous study among soldiers that also relied on administratively-recorded sex crimes [28]; they diverge from another study that used self-reported data to identify sexual perpetration, which did not find any significant differences in race, ethnicity, or educational attainment by perpetration status [27]. Taken together, these findings indicate that these demographic characteristics may be more related to charges and convictions of sexual offenses, rather than perpetrations of sexual offenses. It is also possible that those who have not received a college education may be less sophisticated about circumnavigating the system and hence may be more likely to be charged and convicted of a sex crime. In addition, there are racial and ethnic disparities in the U.S. criminal justice system, where people of color or those who identify as American Indian, Black non-Hispanic, or Hispanic/Latino are more likely to be arrested [48, 49], and this bias likely exists within the U.S. military justice system as well. A 2019 U.S. Government Accountability Office report [50] found racial and ethnic disparities within the military criminal investigative organizations. Given growing concerns of unconscious bias within civilian and military criminal and law enforcement sectors, these potential inequalities in the military law enforcement and justice system may need to be further examined.

Previous studies suggest that additional factors (e.g. early sex, criminal history, substance abuse) may be associated with sexual perpetration [22, 28]. In a civilian sample, engaging in early sex was a predictor of self-reported sexual perpetration [22]; a criminal history and substance use diagnoses and treatment during military service were related to cases of sexual assault among U.S. Soldiers [28]. The current study did not find sexual encounter onset, history of arrest, or pre-service alcohol use predictive of a sexual offense conviction. However, the study samples, assessment of predictors, and type of outcomes varied across these studies. Thus, caution must be taken when comparing results across various studies; in addition, replication and expansion of these type of analyses are needed.

Notably, to our knowledge, this is the first study, that has examined predictors of sexual perpetration among U.S. Marines. This study used pre-service self-reported data to assess potential predictors of subsequent sexual offense conviction among a large cohort of Marines, which allowed for the longitudinal assessment of risk. Test-retest reliability analyses found RAP survey data to be reliable [51]. The outcome was derived from official criminal records, which avoids potentially influential self-reporting bias. However, it is important to interpret the current study findings in light of its limitations. Multiple factors known to be associated with sexual assault perpetration were not assessed as part of the RAP survey (e.g., prior sexual assault perpetration, number of sex partners, and interpersonal hostility). Low rates of sexual assault convictions limited the power of our analyses to identify significant predictors, requiring larger effect sizes to achieve statistical significance and preventing the opportunity to parse the outcome by specific sex crimes. While the overall response rate to the RAP survey is high, this study was restricted to male Marine who trained at MCRD, San Diego; female Marines were not training at this location and data were not collected at MCRD, Parris Island during the study period. MCRD, San Diego also primarily trains those with a home residence west of the Mississippi River, who may be demographically different from those who train at MCRD, Parris Island. The predictors assessed in this study relied on self-reported data, which may be subject to recall and reporting bias.

Even though multiple programs within the DoD have been implemented with the intention of preventing incidents of sexual assault, findings from this study and others suggest that more changes are needed to prevent sex crimes, hold perpetrators accountable, decrease barriers for reporting, and increase support for survivors [52]. Our study findings confirm that some demographic characteristics and factors related to family history, childhood experiences, and risky behaviors present an increased risk for a sexual offense conviction among U.S. Marines. It is noteworthy that events that have occurred in the past continue to influence outcomes for personnel during their military service. Effective prevention efforts could benefit from providing confidential supportive assistance to those who have a family or individual background that may present elevated risk. There are efforts underway that use computer-assisted prevention programs to confidentially assess personnel and provide tailored intervention based on known risk factors. Specifically, the Research Triangle Institute is collaborating with the DoD to provide and evaluate a technology-based, tailored sexual violence prevention training to Air Force Cadets [53]. This program seeks to prevent first-time victimization, re-victimization, and sexual misconduct by combining group classroom instruction with tailored, computer-delivered content. Findings from the current study emphasize the potential of technology-facilitated programs that provide private, targeted education; supportive assistance; and prevention materials to individuals based on risk factors. In addition, understanding how these factors influence an offender’s thinking pattern may help law enforcement investigators identify offending motivation and a pattern of offending behaviors. Thus, study results may also assist investigators in developing interview questions that will give them insight into the lives of service members accused of sexual misconduct.

Further, it is essential to continue research that helps better understand sexual assault and convictions of sex crimes in the military; future research may include examining predictors of sexual misconduct among other service branches and investigating the impact of making a restricted or unrestricted report. There have been additional efforts at the national level to further strengthen the investigation and prosecution of reported sexual assault incidents [45]. Specifically, the 2022 National Defense Authorization Act takes the prosecution of sexual assault away from the chain of command and places it under the purview of a service-specific Lead Special Trial Counsel. This counsel is independent of either the accused or potential victim of the crime’s command. These policy changes can assist in bringing more service members accused of sex crimes to court-martial, possibly increasing accountability and prosecution. Executive Order 14062 of January 26, 2022 also modifies the Uniformed Code of Military Justice to create a separate crime category for sexual harassment, increasing the visibility, and perhaps the prosecution, of this type of crime. These policy changes, in addition to other programs already in place, may decrease institutional barriers in reporting and prosecuting sex crimes. Fewer reporting barriers and more prosecution of sex crimes could in turn discourage sexual harassment and sexual assault in the military. Additionally, these military programs and research in this area may provide vital information that is translatable to civilian settings. Ongoing evaluation of the impact of policy changes and implemented programs will be essential to continue to address this persistent problem of sexual assault in the military.
